# Multicentre randomised controlled trial of a group psychological intervention for postnatal depression in British mothers of South Asian origin (ROSHNI-2): study protocol

**DOI:** 10.1192/bjo.2021.1032

**Published:** 2021-12-01

**Authors:** Nusrat Husain, Karina Lovell, Carolyn A. Chew-Graham, Farah Lunat, Rebecca McPhillips, Najia Atif, Saadia Aseem, Jasmin Begum, Penny Bee, Kamaldeep Bhui, Peter Bower, Traolach Brugha, Nafeesa Bhatti, Nasim Chaudhry, Linda Davies, Nadeem Gire, Anharul Islam, Joe Kai, Jill Morrison, Naeem Mohmed, Jyothi Neelam, Atif Rahman, Shanaya Rathod, Najma Siddiqi, Sadia Shah, Tinevimbo Shiri, Waquas Waheed, Ilyas Mirza, Chris Williams, Nosheen Zaidi, Richard Emsley, Richard Morriss

**Affiliations:** Division of Psychology and Mental Health, University of Manchester, UK; Division of Nursing, Midwifery and Social Work, University of Manchester, UK; and Greater Manchester Mental Health NHS Foundation Trust, UK; School of Medicine, Keele University, UK; Research and Development, Lancashire & South Cumbria NHS Foundation Trust, UK; Social Care and Society, Division of Population Health, Health Services Research and Primary Care, University of Manchester, UK; Perinatal Mental Health, Human Development Research Foundation, Pakistan; Research and Development, Lancashire & South Cumbria NHS Foundation Trust, UK; Research and Development, Barnet Enfield and Haringey Mental Health NHS Trust, UK; Division of Nursing, Midwifery and Social Work, University of Manchester, UK; Centre for Psychiatry, Barts and The London School of Medicine & Dentistry, UK; Centre for Primary Care and Health Services Research, University of Manchester, UK; Department of Health Sciences, University of Leicester, UK; Research and Development, Lancashire & South Cumbria NHS Foundation Trust, UK; Pakistan Institute of Living and Learning, Dow University of Health Sciences, Pakistan; Division of Population Health, Health Services Research & Primary Care, University of Manchester, UK; Research and Development, Lancashire & South Cumbria NHS Foundation Trust, UK; Research and Development, Lancashire & South Cumbria NHS Foundation Trust, UK; School of Medicine, University of Nottingham, UK; Senate Office, University of Glasgow, UK; Research and Development, Lancashire & South Cumbria NHS Foundation Trust, UK; Research and Development, Lancashire & South Cumbria NHS Foundation Trust, UK; Institute of Life and Human Sciences, University of Liverpool, UK; Faculty of Science, University of Portsmouth, UK; Department for Health Sciences, University of York, UK; Research and Development, Lancashire & South Cumbria NHS Foundation Trust, UK; Liverpool School of Tropical Medicine, UK; Centre for Primary Care and Health Services Research, University of Manchester, UK; Adult Mental Health Services, Barnet, Enfield and Haringey Mental Health NHS Trust, UK; Institute of Health and Wellbeing, University of Glasgow, UK; Research and Development, Lancashire & South Cumbria NHS Foundation Trust, UK; Department of Biostatistics and Health Informatics, Institute of Psychiatry, Psychology & Neuroscience, King's College London, UK; Institute of Mental Health, University of Nottingham, UK

**Keywords:** Perinatal psychiatry, depressive disorders, cognitive–behavioural therapies, psychosocial interventions, randomised controlled trial

## Abstract

**Background:**

In the UK, postnatal depression is more common in British South Asian women than White Caucasion women. Cognitive–behavioural therapy (CBT) is recommended as a first-line treatment, but there is little evidence for the adaptation of CBT for postnatal depression to ensure its applicability to different ethnic groups.

**Aims:**

To evaluate the clinical and cost-effectiveness of a CBT-based positive health programme group intervention in British South Asian women with postnatal depression.

**Method:**

We have designed a multicentre, two-arm, partially nested, randomised controlled trial with 4- and 12-month follow-up, comparing a 12-session group CBT-based intervention (positive health programme) plus treatment as usual with treatment as usual alone, for British South Asian women with postnatal depression. Participants will be recruited from primary care and appropriate community venues in areas of high South Asian density across the UK. It has been estimated that randomising 720 participants (360 into each group) will be sufficient to detect a clinically important difference between a 55% recovery rate in the intervention group and a 40% recovery rate in the treatment-as-usual group. An economic analysis will estimate the cost-effectiveness of the positive health programme. A qualitative process evaluation will explore barriers and enablers to study participation and examine the acceptability and impact of the programme from the perspective of British South Asian women and other key stakeholders.

Postnatal depression is a major public health concern because of its negative effect on mothers, including relationship problems^1,2^ and an increased risk of future depression. In the UK, postnatal depression is more common in British South Asian women than White women.^1,3^ Postnatal depression also negatively influences the psychological and cognitive development of children.^2^ Postnatal depression is treatable,^4,5^ but women can be reluctant to disclose symptoms and seek help because of stigma.^6,7^ Antidepressants can be an effective treatment, but new mothers can be reluctant to use them because of concerns about side-effects.^4,5^ Cognitive–behavioural therapy (CBT) has been shown to be effective for postnatal depression^4,5^ and is endorsed as a first-line treatment.^8^ However, there is little empirical evidence for the adaptation of evidence-based treatments, including CBT, to ensure their applicability to different ethnic groups.^9,10^ There is value in cultural adaptation and a need to test the effectiveness of adapted interventions for ethnic minority groups.^11^

## The need for culturally adapted CBT

The positive health programme (PHP) is a culturally adapted group CBT intervention for postnatal depression^10^ that has been developed by our research group in an exploratory randomised controlled trial (RCT) (Exploratory RCT of a Group Psychological Intervention for Postnatal Depression in British Mothers of South Asian Origin; ROSHNI-D),^12^ and we were able to show the feasibility and acceptability of the present trial. A sufficiently powered trial is needed to confirm the preliminary findings of ROSHNI-D, to test both short- and longer-term outcomes, and to answer additional research questions, including the cost-effectiveness of PHP. The focus of the proposed study is a group CBT intervention for British South Asian women with postnatal depression and psychosocial difficulties. These women often live in low-income areas and, because of their psychosocial problems, language and cultural barriers, they are deemed ‘hard to reach’ and hard to engage. South Asian people comprise the largest ethnic minority group in the UK, representing around 7.5% (4 214 000) of the population,^13^ and have one of the highest birth rates in the UK. There are now policies that endorse the need for a range of effective therapies, including psychotherapeutic treatments, that are culturally appropriate and effective.^14^

## Aims and hypothesis

The present trial, ROSHNI-2, aims to evaluate the clinical and cost-effectiveness of the PHP intervention added to treatment as usual (TAU), compared with TAU only, in British South Asian women with postnatal depression. *Roshni* means light in Urdu/Hindi. The primary hypothesis is that participants who receive PHP added to TAU will have reduced levels of postnatal depression, as measured by the Hamilton Rating Scale for Depression (HRSD),^15^ compared with participants receiving TAU only, 4 months after randomisation (end of intervention). HRSD scores at 4-month follow-up will be the primary outcome measure. Costs associated with health and social care resource use will be collected, and each participant in the trial will be asked to complete the EuroQol EQ-5D instrument at each follow-up, and these responses will be converted to quality-adjusted life years (QALYs). Follow-up at 12 months will provide evidence of long-term outcomes. A qualitative process evaluation will explore barriers and enablers to study participation, and examine the acceptability of PHP from the perspective of British South Asian women, the impact of PHP on healthcare practice from the perspectives of general practitioners (GPs), and the experiences of being trained in and delivering PHP from the perspectives of group facilitators.

## Method

### Design

This is a multicentre, two-arm, rater-blinded, partially nested RCT with post-intervention (4 months) and 12-month follow-up, comparing PHP and TAU with TAU only, for British South Asian women with postnatal depression. There is a likelihood that because of the reciprocity between participants during the group PHP intervention, they will respond in a similar manner and their outcomes will be more alike than for women in different groups. This leads to a clustering effect in the intervention condition similar to that found in cluster randomised trials. The trial design adopts a partially nested design with participants in the intervention arm nested in therapy groups compared with the control arm participants, who are not nested. Economic and process evaluations are also embedded in the trial. The trial is registered with Clinicaltrials.gov under registration number ISRCTN10697380.

### Internal pilot phase

An internal pilot phase is integrated into the trial, with clear STOP/GO criteria to prove viability to proceed to a full trial across all study sites. Success criteria during the internal pilot study phase (months 5–14) will be gauged by recruitment of at least 200 participants. In case this is not possible and we face difficulties in achieving the target, we will look into the possible shortcomings and have a ‘rescue plan’ ready for deliberation by the Trial Steering Committee and National Institute for Health Research approval.

STOP/GO criteria are as follows: GO if ≥180 participants are recruited into the study (target minus 10%), implement rescue plan if ≥120 (60%) but <180 (90%) participants are recruited into the study, and STOP if <120 (60%) participants are recruited into the study.

### Setting

Participants are recruited from general practices, children's centres and other appropriate community venues in areas with a high South Asian population across different locations in the UK (North-West, Yorkshire, Midlands, London and Glasgow). Local principal investigators lead the conduct of the trial in each location, and are supported by the ROSHNI research team, all of whom, including principal investigators, are trained in good clinical practice.

### Study population

Participants are women of self-ascribed British South Asian origin who live with their infant(s) aged ≤12 months, have postnatal depression and will reside in the geographical area of the study for the duration of their participation.

#### Inclusion criteria

The inclusion criteria are as follows: self-ascribed British South Asian women as mentioned by the Office of National Statistics,^13^ aged 16 years or older; living with their infant(s), who are aged up to 12 months; and meet the DSM-5 criteria for depression as confirmed by the Structured Clinical Interview for the DSM (SCID).^16^

#### Exclusion criteria

The exclusion criteria are as follows: diagnosed physical or intellectual disability that prevents participation in the trial, postpartum or other psychosis, or active suicidal ideation.

The exclusion criteria will not include ongoing use of antidepressant medication and earlier self-reported common mental illness, including prior postnatal depression. Medication use will be recorded in the baseline and follow-up assessments, and this information will be considered at analysis.

### Recruitment

Potential participants will be approached via GPs, children's centres, baby clinics, community centres, pharmacies and other relevant locations. The study is promoted in each of these locations, using materials that have been produced as a joint collaboration with the patient group, and appear in English, Urdu, Hindi, Bengali, Gujarati and Tamil. Community contact has already been initiated through partnerships with voluntary sector organisations, and the research team has started regular liaison meetings with the local health visitors, GPs and children's centres, for study promotion. Specific inputs are also being taken from the community organisations related to trial design, recruitment methods and the intervention content.

Participating GPs send a letter to all 6-week postnatal British South Asian women informing them about the trial and inviting them to take part. The letter includes the Patient Health Questionnaire 9 (PHQ-9),^17^ a participant information sheet (PIS) and a response slip. Potential participants are asked to complete the PHQ-9 and consent-to-contact form, and return these to their local study site via pre-paid envelope. Postal and telephone reminders are sent to those who do not respond. In addition, GP records are monitored for non-responders so that primary care staff can remind women about the study at routine appointments. When the PHQ-9 and consent-to-contact forms are completed and returned, the research assistants will then make contact and arrange to speak to the participant in a convenient location, to provide further detail on the study and take consent.

Potential participants are also identified by the research team via GP case records. With the GP's permission, identified mothers are contacted either directly by phone or in person, by a clinical support officer, research nurse or GP administration staff, with the support of a bilingual member of the research team. To further ensure efficient recruitment, the research team visits local children's centres, baby clinics and other relevant locations, where potential participants are invited to take part and are provided with a PIS. Women who fulfil trial eligibility criteria are invited to provide written informed consent for trial participation and baseline assessment. All participants will be given ample opportunity for clarifications and time to consider before signing the consent form. The research team will advise participants that their participation in, or departure from, the trial does not affect any aspect of their usual care. Once a patient has agreed to participate and signed a consent form, they are then asked to complete the PHQ-9. Participants who score ≥10 are eligible to progress to the second stage of assessment of eligibility. Mothers who score <10 on the PHQ-9 at first screen will be again invited for a further assessment after 12–16 weeks to complete the PHQ-9 again, if within the 52-week postnatal period, to ascertain that later-onset postnatal depression is not missed.

The second stage of recruitment/assessment of eligibility is an interview with the SCID.^16^ This is administered by trained members of the research team at baseline to confirm the diagnosis of depression. The SCID is a semi-structured interview that consists of standardised diagnostic questions arranged in modules corresponding to each DSM-5 Axis.^16^ The SCID has been used in a large RCT of psychological intervention for postnatal depression with a multicultural population in Canada.^18^ The PHQ-9 has been translated and validated in Urdu.^19^ In cases where the SCID confirms depression, women are asked to confirm consent to take part in the study. In cases where the SCID does not confirm depression, these women are not randomised.

Our previous experience of research with British South Asian mothers who have postnatal depression indicates that engagement with participants’ families is fundamental to success, and that this should begin at the time of screening. Therefore, at the potential participant's discretion, researchers will share details of the study with other family members. Contact details will also be provided for a bilingual senior investigator who will be available to converse with the family if needed. The first study participant was recruited on 8 February 2017.

### Randomisation

Participants are randomised via an independent remote telephone randomisation service based at the Manchester Clinical Trials Unit. PHP is based on groups of between five and nine participants, and randomisation is stratified by centre and will use a block size of 10–18, depending on the size of the site, so that five to nine participants are allocated to PHP plus TAU and five to nine participants are allocated to TAU only. All efforts are made to ensure that the time period between baseline assessment and randomisation will be no longer than 4–6 weeks. The GP is informed about their patient's participation in the trial and treatment allocation by a letter, to be kept with the participant's medical notes.

It is not possible to blind participating mothers, their GPs or practice health visitors to treatment group. Researchers who are blind to treatment allocation will collect the outcome measures. To minimise selection bias and maximise external validity, we will recruit mothers across geographies in the UK and aim to keep the exclusion criteria to a minimum.

### Intervention

PHP is a manual-assisted, culturally adapted, group psychological intervention delivered by two trained, bilingual facilitators (one lead facilitator and a co-facilitator). PHP is based on the principles of CBT, and has been found to be acceptable by participants in previous studies of postnatal depression in British South Asian women.^10^ In ROSHNI-2, 12 group PHP sessions are delivered in total; sessions are delivered once a week for the first 2 months, and then fortnightly for the following 2 months. Each group session lasts 60–90 min, and at the start of each session participants are reminded about confidentiality and are assured by facilitators that any disclosures they make will remain confidential, with the exception of any intentions of harming oneself or others. The sessions covered within the PHP include identifying the pressures and expectations of being a South Asian woman, understanding and managing self-esteem, ‘keeping up with the Chaudhry's’, exercise and looking good, religion and spirituality, relaxation and ‘taking time out’, assertiveness and confidence building, breaking social isolation and building social networks, and practising CBT techniques and assessing change and developing relapse prevention plans.

The processes through which PHP was culturally adapted are described in detail elsewhere.^10,12^ The PHP manual is available in English, Urdu, Hindi, Bengali, Gujarati and Tamil. Before each group starts, facilitators are matched to groups according to languages most commonly spoken by group participants. All facilitators are fluent in English and one or more of the study languages. Our earlier findings point out that when working with diverse populations, the venues where the intervention is delivered should be kept as neutral as possible and ‘not owned’ by a specific ethnic or religious group.^10^ Therefore, PHP is delivered at children's centres and other community venues, as these are considered more acceptable to the British South Asian community than going for ‘therapy’ or ‘treatment’ in a medical setting. Transportation, child care and refreshments are provided to facilitate mothers’ attendance at group sessions. Health Research Authority guidance states that payment to participants in therapeutic research, in addition to reimbursement, are permissible, as they may reduce potential power imbalances by making participation seem less like a favour to a healthcare professional or researcher.^20^ Furthermore, in the UK it is standard practice to pay healthy volunteers and patient volunteers, therefore not paying others who also contribute to research would be unfair, and the benefits that participants may receive from the intervention do not mean that they should be barred from receiving payment. Participants are reimbursed with a £10 ‘love2shop’ voucher for their time and their travel expenses are also reimbursed. The intervention group will continue to receive routine assessment and management by the primary care team. With the written informed consent of participants, a few groups sessions are recorded for quality checks.

### Training, supervision and competency assurance

PHP facilitators are of different backgrounds and have varying levels of experience of delivering psychological interventions in the British South Asian community. Each facilitator received two full days training, which is delivered by PHP trainers who are mental health professionals and therapists by background (K.L., N.A.). The content of each PHP session is covered during training, and time is also spent on non-therapeutic specific skills, including engaging with participants, controlling PHP groups, listening skills and safeguarding. Didactic and Socratic teaching methods are used in PHP training and these are facilitated with presentations, group discussions and role-play methods. At least one out of 12 sessions is observed by PHP trainers, following which constructive feedback is provided and any areas that need improvement are highlighted. Monthly supervision is conducted by the PHP supervisory team and is available between sessions if deemed necessary by PHP trainers or requested by group facilitators. Supervision is conducted over the phone or via Skype in most instances, to account for the geographical distribution of groups and facilitators.

### Treatment as usual

TAU includes participating GPs carrying out routine assessment and management and may involve ongoing monitoring, Improving Access to Psychological Therapies/other services referral and antidepressant prescription.

### Outcome measures

**Fig. 1:**
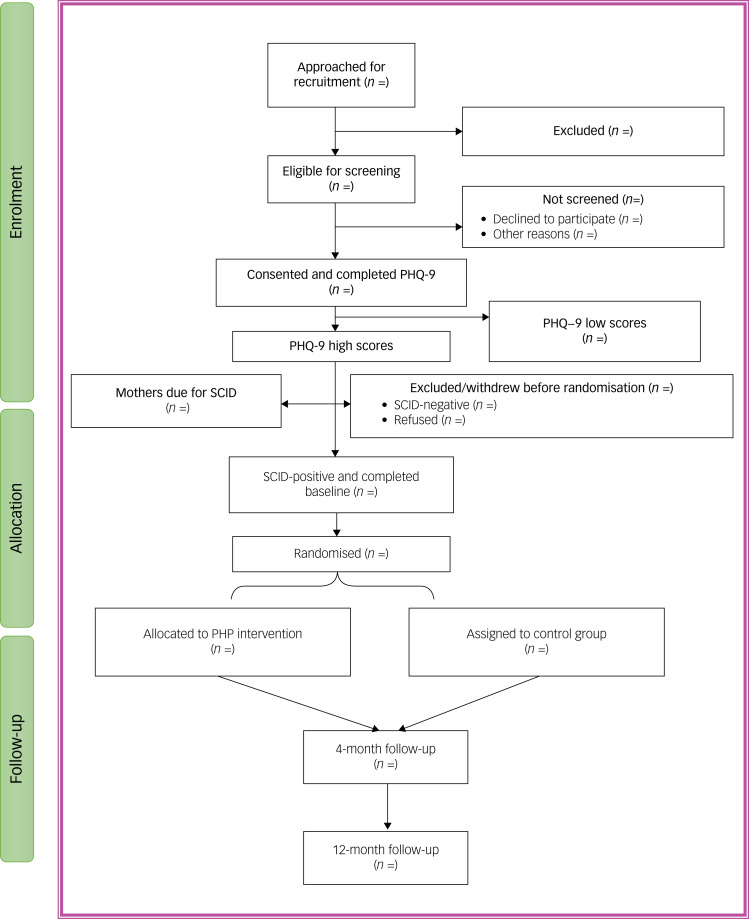
Consolidated Standards of Reporting Trials study flow diagram.

Outcome measure data is collected at two time points: the end of intervention at 4 months, and 12 months after randomisation. The SCID is not repeated at the 4-month follow-up as it is used only as a diagnostic measure in ROSHNI-2, to establish diagnosis of depression (Table 1 and Fig. 1).

**Table 1:** Schedule of assessments

Procedures	Visits (insert visit numbers as appropriate)			
	Screening	Baseline	4 months (end of intervention)	12 months
Informed consent	X			
Demographics		X		
Socioeconomic data		X		
Study assessments				
PHQ-9		X	X	X
HRSD		X	X	X
SCID		X		
GAD-7		X	X	X
EQ-5D		X	X	X
Parenting Sense of Competence scale		X	X	X
Social functioning		X	X	X
IAPT Healthy Minds Patient Experience Questionnaire			X	X
EPQ		X	X	X
Ages and stages questionnaires		X		X

PHQ-9, Patient Health Questionnaire 9; HRSD, Hamilton Rating Scale for Depression; SCID, Structured Clinical Interview for the DSM; GAD-7, Generalized Anxiety Disorder-7: IAPT, Improving Access to Psychological Therapies; EPQ, Economic Patient Questionnaire.

### Primary outcome measure

At the 4-month follow-up, the primary outcome measure is the HRSD,^15^ which is a valid and reliable measure of severity of depression. A score of ≤7 on the HRSD will indicate remission/recovery from depression.

### Secondary outcome measures

Secondary outcome measures are as follows:

- Treatment response will also be measured with the HRSD; treatment response criteria is a reduction of ≥50% in the HRSD baseline score of the participants.
- PHQ-9^17^: valid and reliable self-report measure of severity of depression; Urdu and Bengali versions of the PHQ-9 have been validated.^21^
- Generalized Anxiety Disorder-7 (GAD-7)^22^: a valid and reliable self-report measure of severity of anxiety; an Urdu version of the GAD-7 has been validated.^23^
- Parenting Sense of Competence^24^: a valid and reliable self-report measure of parental competence;^25^ Parental competence is measured on two dimensions of satisfaction and efficacy; the questionnaire has 16 items (nine on satisfaction and seven on efficacy) and is scored according to a six-point Likert scale, ranging from ‘1, strongly disagree’ to ‘6, strongly agree’.
- Social functioning^12^: a self-report measure rating difficulty in completing ten items concerning daily function; the questionnaire is scored according to a five-point Likert scale, ranging from ‘0, no difficulty’ to ‘4, often can't do the task’.
- Improving Access to Psychological Therapies Patient Experience Questionnaire^26^: a valid and reliable self-report measure of an individual's experiences of the intervention they participated in.
- Ages and stages questionnaires^27^: a valid and reliable parent-completed measure of childhood development; this series of 11 questionnaires each include 30 closed questions on five domains of child development; mothers will finish the questionnaire both at baseline and 12-month follow-up.
- EQ-5D-3L:^28,29^ a health status measure, which has five dimensions (mobility, self-care, usual activity, pain/discomfort, anxiety/depression); participants can rate themselves as having no problems, some or moderate problems or extreme problems (for pain/discomfort and anxiety/depression domains), or unable to do an activity (self-care and usual activity domains or confined to bed (mobility domain).
- Economic Patient Questionnaire (EPQ): health service use will be captured using an EPQ; in addition to the National Health Service (NHS) and social care, cost to the participant for services will also be collected; if women in either arm of the trial are receiving any other treatment, this information will be captured here.

All measures are available in Urdu, Hindi, Bengali, Gujarati and Tamil.

### Sample size

We will recruit 720 participants and randomise 360 into each arm. With 40 closed groups of nine and 360 controls, the trial will have 90% power to detect a clinically important difference between a 55% recovery rate in the intervention and a 40% recovery rate in the control, assuming an intraclass correlation (ICC) for group treatment of 0.05, a 75% follow-up and a 5% significance level. The sample size calculation and data analysis take into account clustering because of the partial nested design. Statistical methods for the partially nested design have been discussed in other publications.^30^ Peer-reviewed work by scholars evaluating psychological treatments for postnatal depression points out that for the effect to be considered clinically meaningful, there needs to be a 20% absolute decrease in the number of mothers with depression post-treatment, endorsing the need to develop and implement such treatments. On the recommendation of the funding panel, we will be powered to detect a difference of 15% in the primary outcome between the two randomised arms. Our exploratory trial suggests that a 75% follow-up rate can be achieved at 4 months. Assuming this follow-up rate and an initial group size of nine women per group, the mean group size will be 6.75 women.

There is not enough reliable data related to the magnitude of therapy group clustering when evaluating psychological treatments for postnatal depression. Barrowclough et al give 30 estimates of the ICC for a group therapy, ranging from 0 (95% CI 0–0.29) to a maximum of 0.257 (95% CI 0.02–0.67), with a mean of 0.044 and median of 0.^31^ From the width of the confidence intervals, there is appreciable uncertainty regarding these estimates. An ICC of 0.05 approximates to a moderate Cohen effect size (0.5) and this is close to the mean of the estimates from Barrowclough et al.^31^ The study is robust against larger values of the intracluster correlation for group treatment. For example, if the intracluster correlation coefficient is as large as 0.1, power will still be 86.7%. We therefore believe that the value of 0.05 is a reasonable choice of ICC to use for sample size calculation. In some smaller sites, to reduce the wait for participants for PHP groups to start smaller PHP groups with five to six (10–12 SCID-positive) participants may be run. Reducing the group size to an average of five participants and recruiting a larger number of groups would further increase the power to 91%.

### Statistical analysis

We will use intention to treat analysis. Statistical analysis will be carried out with a logistic random effects model to estimate the odds ratio of recovery between treatment arms, with additional covariates of centre, baseline severity of depression and education (<8 years, ≥8 years). The analysis will be used for both the primary outcome measure (recovery at 4 months, end of intervention) and the further outcome (at 12 months). Random intercepts will be included for the therapy groups, with control participants treated as clusters of size 1, using the methods described by Roberts and Roberts^30^ for the analysis of a partially nested trial.

The treatment effect for quantitative secondary outcome measures will be estimated with a similar approach, with a linear random effects model and a random intercept for therapy group. Baseline value of the measure, centre, parity and educational level will be included as covariates. Statistical analyses will investigate baseline factors that predict non-response by using a logistic random effects model, as non-response may be clustered by therapy group. Sensitivity analysis will be carried out under a range of assumptions regarding missing data informed by the analysis of non-response.

### Economic evaluation

#### Measurement of resource use and costs

The primary economic analysis will use the perspective of the UK NHS and personal social services,^32^ and costs will include those associated with use of NHS and social care primary, secondary and community based services. We believe that direct costs are mainly related to the use of in-patient, out-patient and clinic services at the beginning of the trial and related follow-ups. Sensitivity analysis will take a broader cost perspective to include voluntary care services as well as patient and family expenses. An EPQ data collection tool developed by the applicants will be used to collect service use data. A similar form has been used in an earlier completed trial of complex behavioural and psychological interventions in mental health.^33^ All participants will be asked to complete the EPQ at enrolment and every follow-up assessment. Details to be collected include whether the participant has been admitted to psychiatric or non-psychiatric in-patient care or whether they used out-patient hospital services, including the names of the hospitals attended. The gathered information will be used to identify the relevant hospitals and patient records to extract more details of each admission and resource use by the participants. The EPQ will also ask for other non-hospital based health and social care and third sector services use by participants. Information related to any additional support from faith healers and Imams, and any out-of-pocket spend, will also be collected.

To calculate the cost of each component, national unit costs will be used and each item of resource use will be multiplied by the unit cost specific to that item. Hospital services will be costed by using the relevant national reference costs for each type of admission data published annually by the Department of Health.^34^ Unit costs for medications will be obtained from the British National Formulary. Other sources of unit costs for other services will be based on unit costs of health and social care published annually by the Personal Social Services Research Unit, University of Kent.^35^

#### Measurement of health outcomes

The measure of health benefit for the primary economic analysis will be QALYs estimated from the EQ-5D-5L. The EQ-5D-5L is a validated, generic health status measure, designed to compare health outcomes across diseases. The National Institute for Health and Care Excellence recommends the QALY and the EQ-5D-5L as measures for economic evaluations.^32^ The EQ-5D-3L responses will be used to generate utility values for each assessment. The economic analysis will use the value set for the UK general population, since alternative value sets relevant to South Asian populations are not available.^36^ The utility scores for each participant from baseline to the end of follow-up will then be used to calculate QALYs as follows:

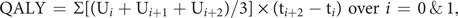

where U is utility and *t* is time at assessment. The time between assessments is the time from baseline to 12-month follow-up (*i* = 2).

#### Cost-effectiveness analyses

The cost and health benefit data will be analysed by treatment allocated and include data for all participants whether or not they completed planned care. However missing data are inevitable from loss to follow-up or missing observations. Single imputation will be used for missing baseline measures of cost, utility and clinical indicators,^37^ but not missing demographic data. An indicator for missing demographic data will be used.

The final imputation strategy will depend on the pattern of missingness in the data. If the data approximate missing at random or missing completely at random, multiple imputation from available data will be used, as recommended by Faria et al.^38^ Literature review and regression analysis of pooled data (masked to treatment allocation) will be used to identify key baseline and follow-up variables associated with costs and QALYs to include in the imputation models. The data will be imputed by category of cost and EQ-5D domain to make best use of available data.

#### Primary analysis

Descriptive analyses of costs and summary health benefit measures will be reported for the intervention and comparator for the complete case and imputed data at baseline and follow-up assessment points. Regression analysis will estimate net costs and QALYs of the intervention. Covariates to account for factors that influence costs or QALYs will be identified from published literature, supplemented with analysis of pooled baseline data.

The regression-based estimates of costs and outcomes will be bootstrapped to replicate 10 000 pairs of incremental cost and QALY outcomes of the intervention. The distribution of pairs of net costs and QALYs will be plotted on the cost-effectiveness plane.^32^

Incremental cost-effectiveness ratios estimate the net cost per QALY gained by an intervention, and raise the question whether that cost is worth paying. To address this, the ICERs are compared with how much decision makers may be willing to pay for an additional QALY. However, the UK has no universally agreed cost-effectiveness threshold. The National Institute for Health and Care Excellence^39^ has previously suggested a threshold of £20 000 to £30 000 per QALY. However, other commentators suggest this may be lower.^40,41^ Reflecting this lack of consensus, the monetary value of simulated QALYs will be estimated across the range of £0–£30 000 willingness-to-pay thresholds. This recognises that decision makers may not be willing to pay for an additional QALY (in other words, they may only seek the lowest cost option) or could be willing to pay up to £30 000 for an extra QALY gained. To estimate the likelihood the intervention is cost-effective for the primary analysis, a willingness-to-pay threshold of £15 000 (the mid-point of the £0–£30 000 range) will be used.

Cost-effectiveness acceptability curves will be generated to show whether the likelihood that the intervention is cost-effective at each willingness to pay value is above 50%. As decision makers increase what they are willing to pay for an extra QALY, the additional health benefits from an intervention become more valuable, and it achieves net benefit in a bigger proportion of the 10 000 replicates.

### Sensitivity analyses

Sensitivity analyses will be conducted to assess the effect of structural uncertainty introduced by the design of the economic evaluation. These analyses include re-estimating the results, using the primary clinical measure as the measure of health benefit; different approaches to missing data, such as use of complete cases only, or indicator values; broader cost perspective; and predictions of costs and QALYs beyond the 1-year trial horizon (for example, over longer time horizons of 5 and 10 years).

### Qualitative process evaluation

Although RCTs are the gold standard for establishing the effectiveness of complex interventions such as PHP, quantitative data does not provide insights into how complex interventions might be replicated or insights into how these interventions are experienced by patients. Process evaluations investigate the ‘reach’ of interventions.^42^ Exploring the mechanisms through which interventions bring about change is crucial to understanding how the effects of the specific intervention occurred, and how these effects can inform the development of similar future interventions.^43^

To learn lessons for recruitment and acceptability of the intervention for the full trial, during the internal pilot phase of the trial, we will examine barriers to recruitment and retention and also look at personal experiences of the women who withdrew from participation in the trial. We will invite women and PHP facilitators to participate in a semi-structured interview.

In the full trial (following successful completion of the pilot), we will conduct interviews with women who have completed the intervention to explore acceptability of the PHP, and women who enrolled in the trial but only attended one or none of the PHP sessions. We will interview group facilitators who have delivered the PHP intervention, and GPs who have engaged with, and referred women to, the ROSHNI trial, Semi-structured interviews will be utilised to explore views on the perceived feasibility, acceptability and sustainability of the PHP in the management of postnatal depression, in this patient group, in primary care.

Topic guides have been developed for the pilot phase by the research team following review of the literature and discussion with patient and public advisors, and will be iteratively developed and refined during the pilot and then in the main trial as analysis progresses.

The objectives of the qualitative study are to explore early barriers and enablers to study participation, to optimise ongoing trial recruiter training and trial recruitment rates (pilot); identify reasons for continuing or not continuing with the study (pilot and full trial); examine the acceptability of the group intervention from the perspective of British South Asian women (pilot and full trial); explore views of the GPs on the group psychological intervention and its impact on practice (full trial); and explore perspectives of group facilitators (group psychological intervention deliverers) about training and delivery of the intervention (pilot and full trial).

Participants are given information about the qualitative aspect of the trial during the baseline assessment and also asked for consent to being contacted by the research team to participate in an interview. Perspective interviewees are identified via a purposive sampling strategy, and to achieve the purpose of inviting patients from different socioeconomic backgrounds, ages and native languages, maximum variation sampling techniques are used and patients are sampled from across the five centres for the trial. Interviews are conducted with women once the primary outcome measure has been obtained (4 months post-randomisation) so as to avert bias likelihood that might be introduced by the supportive role of the qualitative interview. Participant interviews will be conducted within 8 weeks of their primary outcome measures being collected.

In the full trial, up to 20 women who participated in the group sessions will be interviewed with the aim of examining at length the acceptability of group meetings, viewpoints on discussing personal problems in front of other participants, views on the involvement of the group facilitator, family's perspective on the participants’ attendance and the effect on the current situation. Interviews will also be conducted with up to 20 women who dropped out of the study after one or no session, to better understand their reasons for not continuing with the sessions.

Interviews will also be conducted with group facilitators (up to 15) to investigate barriers and enablers to the group work. GPs participating in the trial (up to 15, across study sites) will be invited to participate in interviews to further our understanding on the management of British South Asian women with postnatal depression and the impact of the intervention on routine practice work.

The researcher will contact the trial participants, GPs and group facilitators, check consent, and arrange an appointment to interview them at a time and place convenient to the respondents (e.g. home, telephone, general practice). Before the face-to-face or telephone interviews, written consent to participate in the interview will be obtained. Telephone interviews, lasting up to an hour, will be conducted with suitable equipment and infrastructure. The researcher will obtain consent from the participant to record and transcribe verbatim the interview. Where the participant does not speak English as their first language, interviews will be carried out in the participant's preferred language and later translated for analysis. Existing recommendations for conducting and reporting cross-language qualitative research will be used where applicable,^43^ including pilot testing the interview guide translation before carrying out the interview, independent verification of translated interviews, and detailing the role and credentials of the translator in any outputs.

### Data analysis

Thematic analysis with principles of constant comparison^44^ will be conducted. This will be followed by framework analysis, which allows for the inclusion of *a priori* as well as emergent concepts.^45^ Framework analysis is effective for using data collected through open-ended questioning and focusing it toward a particular research question in health services research, particularly when a large body of data has been generated.

There are five phases of the framework method of analysis, which are interlinked to form a rigorous framework: familiarisation with the data; identifying a thematic framework; indexing; charting; and mapping and interpretation. These phases enhance understanding and interpretation of the data from descriptive accounts to a conceptual explanation.^45,46^

The framework analysis will be sensitised by the use of the theoretical framework of acceptability.^47^

Regular meetings between the three researchers (S.A., R.M. and J.B.) who are responsible for independently carrying out data coding, and their supervisors (C.A.C.G. and P.Bee.) will be organised to ascertain that the emerging codes remain grounded in the original data.

### Public and patient involvement and engagement

Patient and public involvement and engagement was of great benefit to the recruitment and retention strategies used during the ROSHNI-D trial, and for dissemination.^12^ As such, patient and public involvement and engagement activity is planned throughout the trial.

Initial discussions with key stakeholders from institutions to hold workshops in temples, gurudwaras, mosques, churches, and children's and community centres were held so that we can further community engagement. Workshops provide information on depression in general, and particularly postnatal depression in British South Asian women. We record and take into account feedback from workshops, with particular focus on how to address stigma. The advisory group members provide a crucial support when it comes to community engagement. The advisory board also engages patients with trained expertise in research methods and have trained over 50 patients and carers in research methods. Seminal work by Lovell and colleagues has a good practice citation by the National Institute for Health and Care Excellence.^48^

### Ethics

The research team brings with it an enriching clinical and research experience, and has hands on training in managing challenging situations in research interventions and interviews. We are cognisant of the fact that some respondents can be vulnerable, therefore we will make sure we provide adequate supervision of the researchers, keeping in mind safeguarding participants’ welfare. Timely and regular supervision will be carried out during the entire course of the project for all research staff and group facilitators, including training the staff in good clinical practice and safeguarding (study ethical approval number: IRAS 187851).

Clear guidelines relating to a distress policy have been formulated, keeping in mind the potential effects of conducting research with this group. A distress policy has been formulated. A number of practical measures are taken to minimize distress and risk to participants; for example, when obtaining consent, researchers clarify to participants that they are able to take time in answering questions.

Monitoring, recording and reporting of adverse events will be managed with the local principal investigator, the participant's GP and health visiting team, and in line with each Trust's policy. The Clinical Trials Unit will carry out audits and safety monitoring. Safety protocols have been designed and developed to ensure that safety of research staff, participants and their families remain safe when making contact, conducting research and afterward. This includes researchers confirming that they are speaking with the participant and not leaving information with other household members, asking participants in qualitative interviews how they are feeling at the end, and signposting relevant support when necessary, and researchers following lone-worker policy.

### Progress with ROSHNI-2 internal pilot

The internal pilot was designed to test the ability of the four additional centres to recruit to time and target, with a target recruitment of *n* = 180−200 patients within a 12-month period (November 2016 to October 2017), with a minimum of 120 patients for the trial to continue. Because of a variety of delays, all sites started late, and thus the recruitment period for the internal pilot centres was extended until June 2018.

Recruitment of more than the minimum 120 patients during the internal pilot up to 30 June 2018 was achieved, and the internal pilot target was met to ensure the study continued recruitment. Following the internal pilot, the decision was made to close one site and the resource saving to be redistributed to capitalise on the greater recruitment opportunity in other recruitment sites. The Glasgow site was not able to recruit sufficient number of participants because of low British South Asian birth rates.

### Summary

This study is unlike other comparable mental health studies or even physical health trials with British South Asian participants, given the stigma of mental health in the different South Asian communities and the difficulty in engaging young women, particularly in households often dominated by men and older people. Each site requires its own culturally adapted approach for community engagement and not just one approach. Despite its challenges, the internal pilot study has been successful in demonstrating the viability of progression to a full trial by ensuring that trial processes relating to recruitment, randomisation, treatment and follow-up assessments now work well. In addition, the value of the internal pilot period has been recognised in reflecting on lessons learned. This allowed us to improve the management, communication, processes and efficiency of all trial activity, thus optimising outputs of all remaining centres as we moved into the full trial.

### Dissemination

The results of the study will be published in peer-reviewed journals and presented at national and international conferences. Study findings will also be disseminated to local and national groups in partnership with patients, carers and local voluntary organisations such as MIND, Ethnic Forum and Social Action Forum. Results will also be publicised through a website, short films and our media partners, including Pendle Radio and Asian Image, and through local temples, mosques, churches and community centres, using our existing networks such as the Black and Minority Ethnic Network Lancashire. Information about the research will also be disseminated to the British Council, which has extensive networks not only within the UK, but also overseas.

We will also take advantage of our expertise in working with the NHS and voluntary sector, and share the training needs and information of the culture specific needs of this group. Extensive consultation rounds related to the rich knowledge gained from the trial will be shared with NHS stakeholders and with relevant local, regional and national policy makers and commissioners, to better inform decision-making and service improvements. Consultations will also include patients from the local communities and trial participants. A low-cost PHP manual and how-to ‘tool kit’ will be a crucial output of the study.

## Data Availability

The data that support the findings of this study will be available from the corresponding author, N.H., upon reasonable request, following the data lock, analysis and publication of findings.
